# Case of a Long-Time Survivor with Recurrent Multiple Brain Metastases from Lung Cancer: Terminal Stage Cancer or Chronic Disease

**DOI:** 10.7759/cureus.8378

**Published:** 2020-05-31

**Authors:** Ming Pan

**Affiliations:** 1 Radiation Oncology, Windsor Regional Hospital Cancer Program, Windsor, CAN

**Keywords:** non-small cell lung cancer, multiple brain metastases, whole brain irradiation, hypofractionated stereotactic radiotherapy, neurotoxicity

## Abstract

Lung cancer with brain metastasis has a poor prognosis and has always been treated with palliative intent in the past. We report a case of good response to two courses of whole brain irradiation (WBI) and one course of hypofractionated stereotactic radiotherapy (HSRT) over a nine-year period, with no significant neurotoxicity.

We present the case of a 43-year-old non-smoker female with biopsy-proven adenocarcinoma of right lower lobe lung, stage T1N2M0, positive for anaplastic lymphoma kinase (ALK). She received concurrent 60-Gy chemoradiation in 30 fractions in 2010. She had no local recurrence, but biopsy confirmed distant metastases in neck lymph nodes nine months later. As a result of a 3-cm brain metastasis in the right cerebellum on computed tomography (CT), she received 20-Gy WBI in five fractions. We did not treat any other distant metastases with radiation. She only received systemic tyrosine kinase inhibitors (TKIs).

Four years after the first WBI, she developed headaches and balance problem. MRI showed at least nine metastatic lesions in the brain. She had a craniotomy to remove the largest 4-cm lesion in the right cerebellum followed by 21-Gy WBI in seven fractions. Her symptoms disappeared, and she went back to her normal life. Another MRI three years after the second WBI showed progression of the largest brain metastases in the right cerebellum. We offered her 20-Gy HSRT in five fractions every other day.

She tolerated HSRT very well, with no neurotoxicity. She was able to walk without a walker or cane. She could manage all her daily activities. On examination, there was no neurological deficit. CT scan nine months after HSRT showed excellent local control.

Stage 4 non-small cell lung cancer with positive ALK can have a good response to TKI, and long survival is possible even with multiple brain metastases. Not all patients will have severe neurotoxicity after multiple courses of WBI. HSRT can be a good alternative treatment to symptomatic brain metastases if craniotomy is not desirable. The acute and late toxicities can be reasonably tolerated. Long-term local control and good quality of life are achievable.

## Introduction

Lung cancer is the leading cause of cancer death in Canada, accounting for 25.5% of all cancer deaths [1]. Stage 4 non-small cell lung cancer (NSCLC) with multiple brain metastases (mbMets) has poor prognosis, with a median survival of only three to four months from the time of diagnosis [2]. They are usually treated primarily with whole brain irradiation (WBI) with or without surgery [3]. The goals of treatment are to minimize toxicity and to maximize both survival and quality of life (QoL) [4].

Craniotomy is usually performed for symptomatic mbMets followed by palliative WBI, which has not been proven to improve survival. WBI therapy with or without stereotactic radiosurgery (SRS) boost for patients with a limited number of brain metastases has been used in phase III clinical trials, as well as SRS with or without WBI in a similar population. Unfortunately, there is usually no significant improvement in overall survival (OS) [5-9].

We report our first case of nine-year survival of a patient with stage 4 NSCLC with recurrent mbMets treated by two full courses of WBI and a course of hypofractionated stereotactic radiotherapy (HSRT) with no significant neurotoxicity.

## Case presentation

**Figure 1:**
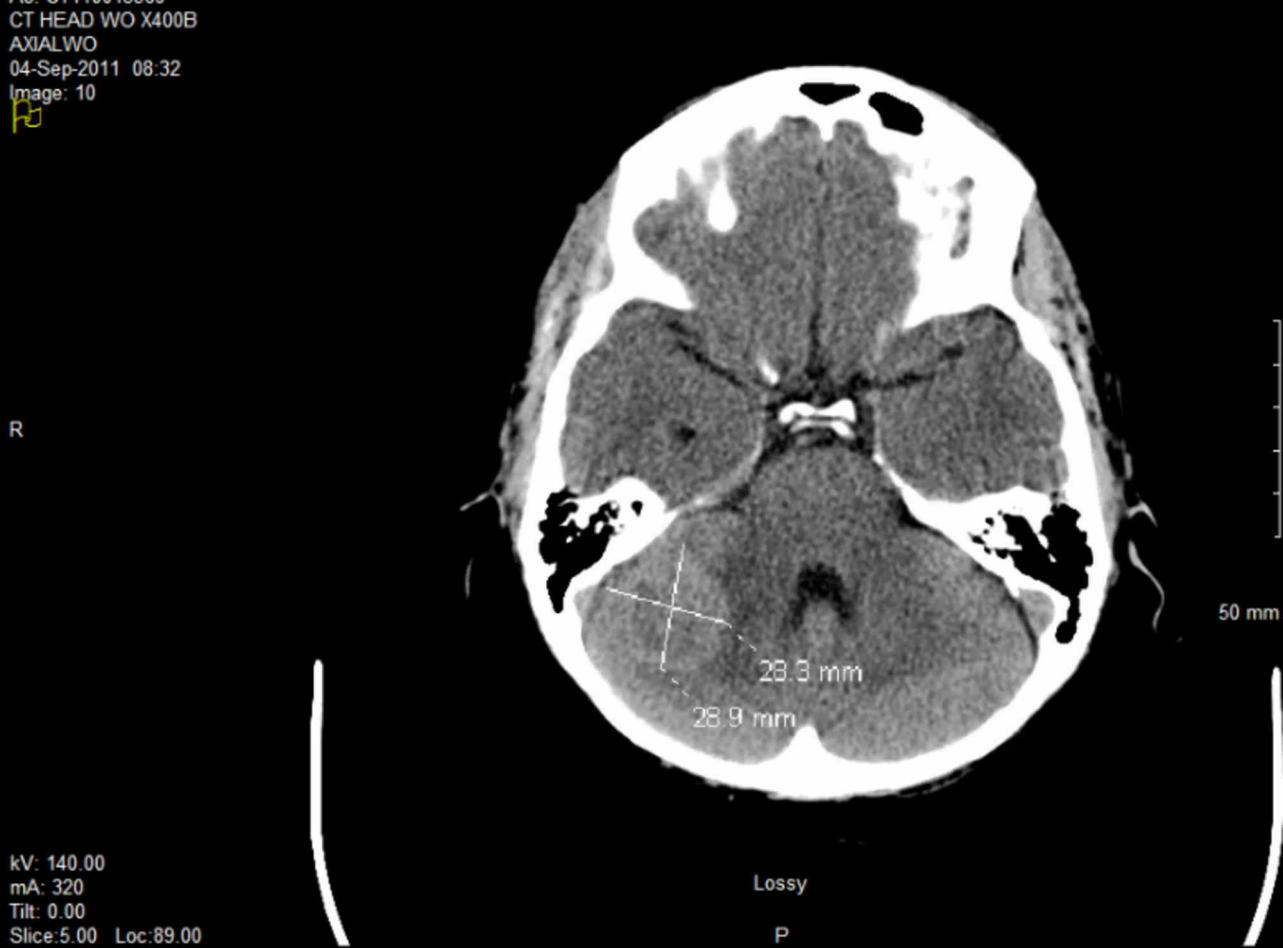
Brain CT showing a large mass in the right cerebellum before the first WBI in 2011 WBI, whole brain irradiation

A 43-year-old non-smoker female presented with biopsy-proven adenocarcinoma of right lower lobe lung, stage T1N2M0 according to the American Joint Committee on Cancer (AJCC) 7th Edition, positive for anaplastic lymphoma kinase (ALK). She received concurrent 60-Gy chemoradiation of in 30 fractions, with the last dose on September 8, 2010. She had no local recurrence, but a biopsy confirmed distant metastases in neck lymph nodes on June 17, 2011. There was also a 3-cm brain metastasis in the right cerebellum on computed tomography (CT) (Figure 1). She received 20-Gy WBI in five fractions. The lesion became much smaller on serial CT scans. We did not treat any other distant metastases with radiation. Surprisingly she did very well with systemic TKIs including crizotinib, ceritinib, and alectinib.

**Figure 2:**
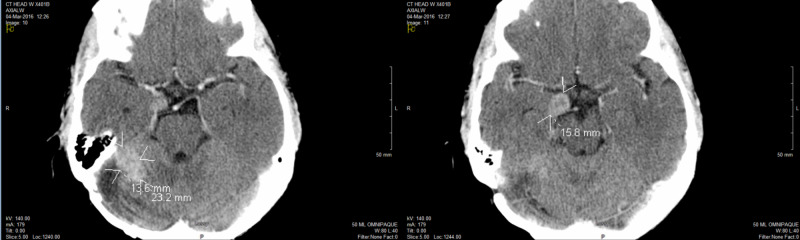
Post-craniotomy brain CT showing recurrent mbMets before the second WBI in 2016 mbMets, multiple brain metastases; WBI, whole brain irradiation

Four years later, she developed headaches and balance problem. MRI on October 30, 2015, showed at least nine metastatic lesions in the brain up to 4 x 3.8 x 2.6 cm in size. She underwent a craniotomy to remove the largest lesion in the right cerebellum on November 23, 2015, and the pathology confirmed lung adenocarcinoma, positive for programmed cell death receptor ligand 1 (PD-L1) 1-49%. Post-operative CT showed multiple residual masses in the brain (Figure 2). She completed another course of 21-Gy WBI in seven fractions on April 19, 2016. Her symptoms resolved, and she went back to her normal life.

Another MRI from May 30, 2019, showed progression of the largest brain metastasis in the right cerebellum measuring 2.5 x 2.9 x 2 cm in size. We offered her 20-Gy HSRT in five fractions every other day to this lesion, which completed on July 26, 2019. She was then started on another TKI, lorlatinib, in August 2019.

She tolerated HSRT very well, with no significant side effects. CT scan four months later showed excellent response with the right cerebellum lesion measuring only 1.5 x 1.4 cm. Her ECOG (Eastern Cooperative Oncology Group) score remained at 1. She had no nausea, vomiting, headaches, memory loss, or any other neurotoxicity. She was able to walk without a walker or cane. She could manage all her daily activities. On examination, there was no neurological deficit.

**Figure 3:**
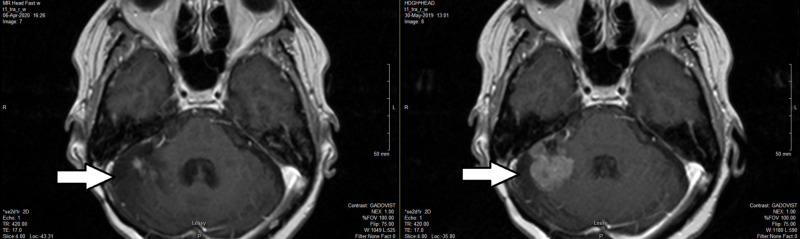
Brain MRI eight months after HSRT (left) versus two months before HSRT (right) HSRT, hypofractionated stereotactic radiotherapy

Her MRI on April 6, 2020, showed a minimal residual enhancement of 0.7 x 0.9 cm in the right superior lateral cerebellum. All the other previously seen intracranial enhancing lesions had almost disappeared (Figure 3).

**Figure 4:**
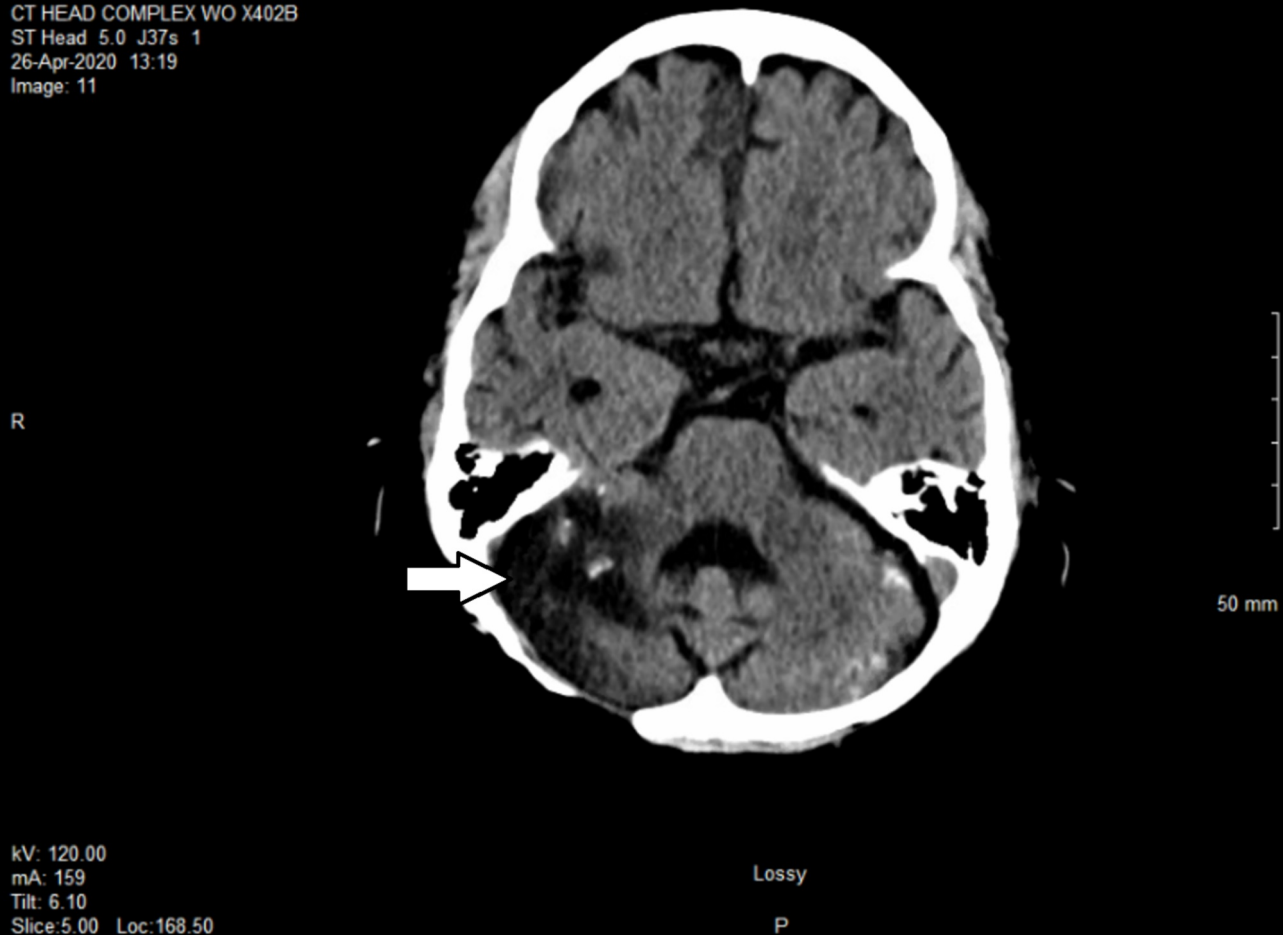
Brain CT showing near-complete response nine months after HSRT HSRT, hypofractionated stereotactic radiotherapy

CT scan nine months after HSRT showed near-complete remission, with innumerable calcified foci in the supratentorial and infratentorial brain in keeping with healed metastases (Figure 4).

Our plan is to follow her closely. Should any of the brain lesions grow much bigger and start to cause symptoms, we will discuss further craniotomy or HSRT to the largest symptomatic lesions. She will continue TKI, with possible PD-L1 inhibitors if there is disease progression.

## Discussion

Lung cancer is a deadly disease. It is estimated that 29,800 Canadians will be diagnosed with lung cancer in 2020 and that 21,200 men and women will die from the disease in the same year [1]. Up to 80% of these lung cancers are NSCLC. Stage 4 NSCLC with mbMets has a very poor prognosis [2]. They are usually treated with WBI with or without surgery followed by palliative chemotherapy or other systemic treatment [3]. The goal is to achieve better local control and QoL [4]. Unfortunately, there is no significant improvement in OS in the past. Radiation oncologists and patients are reluctant to consider multiple courses of WBI due to the concern of severe side effects and worse QoL. Most patients who received WBI reported severe neurotoxicity and never received a second course of WBI or SRS boost. There are rare case reports of long-time survivors, but no cases have been reported for stage 4 NSCLC with recurrent mbMets successfully treated by two full courses of WBI followed by a course of HSRT, with no significant neurotoxicity achieving a nine-year survival.

SRS and HSRT have become popular in recent years, with multiple clinical trials and meta-analysis showing some improvement in survival and QoL [5-9]. Sahgal et al. performed an individual patient data meta-analysis of all phase III trials of SRS with or without WBI for one to four brain metastases (364 eligible patients). For patients ≤ 50 years of age, SRS alone has better OS (p = 0.04). The initial omission of WBI did not impact distant brain relapse rates. Single brain metastasis also had a significantly lower risk of distant brain failure than mbMets with two to four metastases. Local control significantly favored additional WBI in all age groups [10].

Greenspoon et al. examined survival and prognostic factors in a consecutive cohort of patients after the introduction of their SRS program in 2010. Median OS was not significantly different, with 4.3 months in the SRS cohort (167 patients) vs 3.9 months in the pre-SRS cohort (91 patients) (p = 0.74). The result was similar when the no-treatment group was excluded from the SRS cohort. Within the SRS cohort alone, improved OS was seen in patients receiving SRS compared with WBI (p < 0.001), likely due to selection bias [11].

The Radiation Therapy Oncology Group (RTOG) 9508 trial demonstrated survival advantage in the WBI and SRS group versus WBI alone for patients with a single brain metastasis (median OS: 6.5 vs. 4.9 months; p = 0.0393), but not for patients with two or three brain metastases [7]. Rodrigues et al. reported a phase II multi-institutional trial of fractionated simultaneous in-field boost for brain oligometastases. One to three brain metastases received 60-Gy WBI in 10 fractions simultaneously with 30-Gy WBI in 10 fractions at five institutions. Median OS was 5.4 months, and 6-month actuarial estimates of intracranial control (ICC) was 78%, demonstrating non-inferiority to the RTOG 9508 historical controls (OS: p = 0.09; ICC = 0.31) [12].

Our institution started our HSRT program in 2018. We are very diligent in selecting only the most suitable patients with brain metastases [13]. Our current protocol allows up to two brain oligometastases to be treated with an HSRT dose of up to 30 Gy in five fractions every other day. This also includes patient who received 20-Gy WBI in five fractions in the past but developed progression of one or two lesions in the brain.

**Figure 5:**
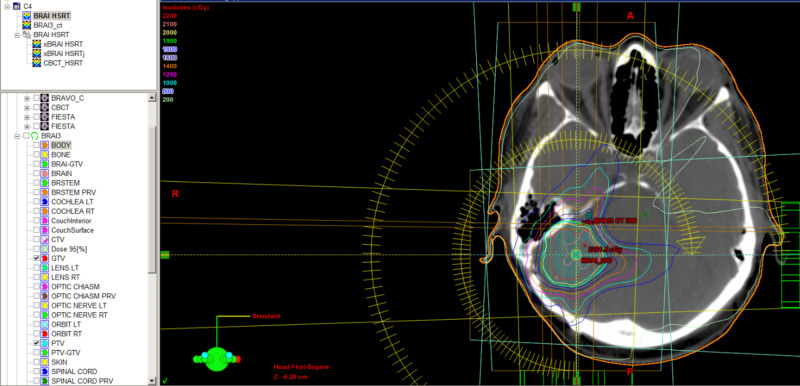
HSRT plan on CT simulation imaging fused with MRI HSRT, hypofractionated stereotactic radiotherapy

**Figure 6:**
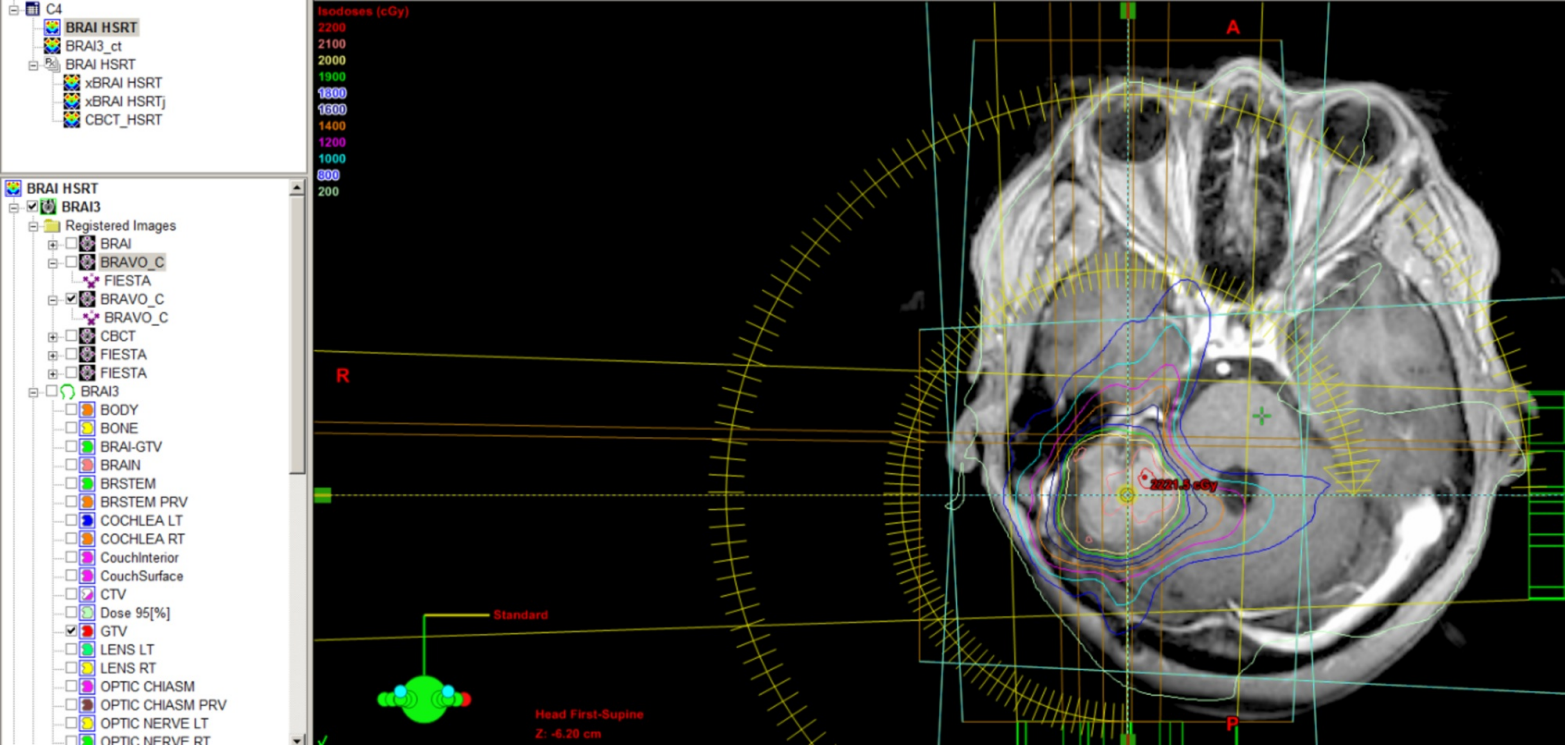
HSRT plan on MRI simulation imaging fused with CT HSRT, hypofractionated stereotactic radiotherapy

Our standard HSRT protocol involves CT and MRI simulation on the same visit, using the same immobilization devices. Medical physicists perform CT and MRI fusion for better contouring of the gross tumor volume (Figures 5, 6). We choose the safest dose fractionation with HSRT every other day. All HSRT treatment delivery is given using image-guided radiotherapy, specifically daily cone-beam CT prior to treatment.

For mbMets, we usually offer WBI alone due to the much worse prognosis as compared with single brain metastasis. If patients survive more than 12 months after the first course 20-Gy WBI in five fractions, we do offer a second course of 21-Gy WBI in seven fractions for symptomatic progression of mbMets. This patient is a surprise to us with excellent response to TKI despite recurrent distant metastases outside of the brain and up to nine brain lesions on MRI. They responded well to two courses of WBI. She was reluctant to consider a second craniotomy when the right cerebellum mass progressed. She was aware of the possible severe neurotoxicity, which never happened in her case, and consented to another 20-Gy HSRT in five fractions. We chose this lower dose due to her previous two full courses of WBI. Fortunately, this achieved excellent local control and we kept her QoL.

It should be noted that we did not treat her other distant metastases outside of the brain with radiation. Her high-dose chemoradiation to the chest was completed before the finding of distant metastases, and therefore this is not a standard palliative approach as recommended in the published guidelines [14].

## Conclusions

Stage 4 NSCLC with positive ALK can have a good response to TKI, and long survival is possible even with mbMets. Not all patients will have severe neurotoxicity after multiple courses of WBI. HSRT can be a good alternative treatment for symptomatic brain metastases if craniotomy is not desirable. The acute and late toxicities are reasonably tolerated. Long-term local control and good QoL are achievable. Aggressive treatment should be considered for highly select patients with good performance status. With modern combined modality treatment, some of the terminal stage cancers might have become a chronic disease.
